# Physical and chemical properties and sensory evaluation of camel meat and new camel meat jerky

**DOI:** 10.1002/fsn3.4310

**Published:** 2024-07-31

**Authors:** Jindi Wu, Xige He, Xueyan Yun, Mei Qi, Buren Menghe, Lu Chen, Yunfei Han, Yajuan Huang, Mingxu Wang, Rina Sha, Gerelt Borjigin

**Affiliations:** ^1^ College of Food Science and Engineering Inner Mongolia Agricultural University Hohhot China; ^2^ State Key Laboratory of Reproductive Regulation and Breeding of Grassland Livestock, School of Life Sciences Inner Mongolia University Hohhot China; ^3^ Alashan Right Banner Ji Xiang Wu Zhen Breeding Herdsmen's Professional Cooperative Alashan China

**Keywords:** camel jerky, formula optimization, lactic acid bacteria, process optimization, quality characteristics

## Abstract

Alxa Bactrian camel meat is an organic diet that provides balanced nutrition and is easy to digest and absorb. Despite its potential, it is currently underutilized. To develop a new type of camel jerky, this study utilized a single‐factor design method to optimize the formula and fermentation process parameters of Alxa Bactrian camel jerky. Additionally, the physical and chemical composition, nutritional components, protein degradation, and microbial changes of the camel jerky were analyzed to evaluate the nutritional benefits of the new camel jerky created using modern fermentation technology. The results show that the optimal addition amounts of camel fermented jerky ingredients are 2.2% black pepper, 3% salt, 1.8% white sugar, and 9% cooking wine, whereas the optimal fermentation conditions by compound starter culture are 0.07% starter, 23 h of fermentation time, and a 30°C fermentation temperature. Compared to the single starter, the compound starter significantly increased the protein content, total amino acids, unsaturated fatty acids, trace elements, and a*, L*, and *e*‐values of jerky; however, it also decreased the water activity (*a*_w_), thiobarbituric acid, and pH values of jerky during storage. Compound starter strains can compensate for the shortage of single starters and improve the overall quality of meat products. These findings could provide new insights and serve as a reference for the development of camel meat products and hold significant importance for the growth of the camel industry.

## Graphical abstract

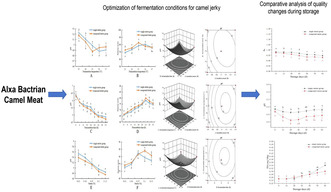

## 1 INTRODUCTION

Camels are a unique species of livestock that inhabit desert and semiarid areas. Since their domestication, camels have been utilized for various purposes, including transportation as well as supplying milk, meat, and wool. The most important camel product is meat, which is a desirable animal food source for humans. Camel meat is an excellent source of high‐quality protein that is low in fat and cholesterol and rich in polyunsaturated fatty acids (Kadim et al., 2013). Studies have shown that camel meat is rich in amino acids, unsaturated fatty acids, iron, calcium, phosphorus, and other minerals; in addition, it is high in moisture and protein as well as low in fat and other components (Kadim et al., 2008). Therefore, camel meat is considered an important raw material for the production of meat products (Kadim et al., 2013). In China, existing camel meat products consist of canned and air‐dried camel meat produced in Alxa using traditional processing methods, such as air‐dried curing and smoking. People's acceptance of camel meat flavors is limited to frying and cooking (Faye et al., 2013; Guya & Neme, 2015). Abdel‐Naeem and Mohamed (2016) improved the physicochemical and sensory properties of camel meat burger patties using a mixture of papain and ginger extract. Gheisari et al. (2008) emulsified camel meat sausage with kiwi flavin, and the results showed that camel meat sausage tasted better than beef sausage. Abdel Kareem (2010) heat‐treated and smoked mature camel meat to develop a variety of new camel meat products.

Jerky is a popular specialty food in Inner Mongolia, China. Studies have shown that during the drying process, the internal moisture of jerky rapidly evaporates through its surface. When the internal water escapes from fresh meat, the meat hardens. However, the fermentation process can improve the texture, impart a distinctive flavor, and boost the nutritional value of the meat (Zhao, Zhao, et al., 2016). In the production of fermented jerky, the flavor and quality of the jerky can be enhanced by adding the appropriate spices during the curing process. Furthermore, the quality of the fermented jerky is affected by the temperature, time, and type and amount of fermenting agent added during the fermentation process (Lorenzo et al., 2014; Tjener et al., 2004; Xiao et al., 2020). In this regard, lactic acid bacteria are commonly used as a fermenting agent in meat fermentation (Ojha et al., 2018).

In the food processing industry, lactic acid bacteria can lower the pH of products, inhibit the growth of other miscellaneous bacteria, prevent spoilage, improve the color of products, and produce unique fermentation flavors (Cardinali et al., 2018; Cruz et al., 2021; Holck et al., 2017). The present functional starter most widely used in fermented meat products in China offers high safety, strong beneficial growth, and good flavor (Long et al., 2016). Different microbial strains have distinct properties; therefore, their effects on fermented meat products differ. Compound starter strains can compensate for the shortage of a single starter and improve the overall product quality. Hu et al. (2019) used a compound starter to produce sausages and discovered that compound starter strains also improve the redness, color, and flavor composition of the sausage while decreasing its moisture content and water activity (*a*_w_).

The Alxa Bactrian camel is a regionally bred Alxa union of desert camels and Gobi camels. They are acclimatized to drought and high and low temperatures and consume desert plants. Moreover, they account for nearly two thirds of the total number of camels in China. They have a very important position in the animal husbandry industry. However, in addition to the service value, the consumption of the Alxa Bactrian camel has not been fully utilized. This study optimized the formula (including black pepper, salt, white sugar, and cooking wine) and fermentation conditions (including amount of starter culture, fermented time, and fermented temperature) of jerky. On the basis of optimizing the formula and fermentation conditions, the fermented camel jerky with single starter culture group and compound starter culture group was prepared. The effects of single and compound starter culture on fermented jerky were studied by measuring the physical and chemical indicators, nutrient compositions, texture, protein degradation, and microbial indicators.

## 2 MATERIALS AND METHODS

### 2.1 Materials

#### 2.1.1 Sample source

A total of 12 male Alxa Bactrian camels were selected from the Alxa Right Banner Ji Xiang Wu Zhen Breeding Herdsmen's Professional Cooperative, Alxa Right Banner, Alxa League, Inner Mongolia, and they were raised under the same conditions (food, water source, and environment). The camels were fasted and drank appropriately before slaughter, and slaughtered at a local abattoir on the same day, in accordance with the Laboratory Animal—Guideline for Ethical Review of Animal Welfare (National Technical Committee on Laboratory Animals of Standardization Administration of China, 2018) and with the approval of the Specialized Committee on Scientific Research and Academic Ethics of Inner Mongolia Agricultural University (Approval Document Number: [2020]002; dated April 7, 2020). Approximately 5 kg of meat from two hind legs was collected, sectioned, and vacuum‐packed for frozen storage.

#### 2.1.2 Starter culture

*Lactobacillus plantarum* JYLP‐326 (single starter) and *Lactobacillus plantarum* JYLP‐19 (compound starter of 45:55, a total of 100%) were provided by Shandong Zhongke Jiayi Biological Engineering Co., Ltd.

### 2.2 Methods

#### 2.2.1 Fermented camel jerky processing

The fascia was removed from the meat. The camel meat was cut into 0.7‐cm meat strips with a uniform thickness. The raw meat was cut into 3.5‐cm‐wide and 8‐cm‐long pieces along the muscle fiber lines. The resulting meat strips were approximately 500 g. The pickling process involved adding peppercorns (0.06%), aniseed (0.06%), and ginger powder (0.06%) with different volumes of black pepper, white granulated sugar, cooking wine, and salt. The starter cultures were washed three times with the same amount of sterile saline solution based on the final level of 10^7^ cfu/g (colony‐forming units per gram) meat. The raw meat and auxiliary materials were rolled and mixed before being placed in a 4°C‐chromatography cabinet. The samples were pickled for 12 h. The required proportion of fermentation liquid was injected into the meat strips after they were cured. The strips were then evenly mixed before being placed in an incubator at 30°C and 90% humidity for 23 h. The fermented meat strips were evenly placed on a drying rack and maintained at 55°C and 70% humidity for 4 h. The meat strips were regularly turned over to ensure even drying. The meat strips were dried for a further 1 h at 45°C and turned over (i.e., every 10 min) to ensure uniform drying. The dried meat was naturally cooled to 25°C before inspection. The best formula and fermentation conditions were used to produce fermented camel meat jerky in the single starter group and the compound starter group, and samples were taken at the control (raw meat), pickled, dried, and mature stages for subsequent index determination.

#### 2.2.2 Single‐factor test and orthogonal test to determine the best camel jerky formula composition

As shown in Table 1, single‐factor and orthogonal tests were performed with black pepper, white granulated sugar, cooking wine, and salt to obtain the optimum basic formula conditions for camel jerky. For sensory evaluation and color difference a* value determination, the factors chosen were the salt levels (1%, 2%, 3%, 4%, or 5%), white granulated sugar (0.6%, 1.0%, 1.4%, 1.8%, or 2.2%), cooking wine (5%, 7%, 9%, 11%, or 13%), and black pepper (1.0%, 1.4%, 1.8%, 2.2%, or 2.6%). After completing all the single‐factor tests, the optimum levels were selected to further determine the formula.

**TABLE 1:** Independent variables in the experimental plan.

Level	Variables			
	A: Black pepper (%)	B: Sugar (%)	C: Cooking wine (%)	D: Salt (%)
1	1.8	1.4	7	2
2	2.2	1.8	9	3
3	2.6	2.2	11	4

#### 2.2.3 Single‐factor experiment for optimal fermentation conditions of camel jerky with an optimization design using response surface methodology

The pH of meat products is an important factor in determining the freshness, taste, and overall quality of meat. The single‐factor and response surface method tests were performed with amount of starter culture, fermentation time, and fermentation times to obtain the optimum fermented conditions for camel jerky. For sensory evaluation and pH value determination, the selected factors were additions of starter of 0.01%, 0.04%, 0.07%, 0.10%, and 0.13%; fermentation times of 12, 16, 20, 24, and 28 h; and fermentation temperatures of 25, 28, 31, 34, and 37°C. Combined with the single‐factor experimental results, a model was established using the Box–Behnken center combined experimental design principle in Table 2.

**TABLE 2:** Values of factors in the Box–Behnken design.

Level	Factor		
	A: Amount of starter added (%)	B: Fermentation time (h)	C: Fermentation temperature (°C)
1	0.07	20	28
0	0.10	24	31
−1	0.13	28	34

#### 2.2.4 Determination of color, *a*_w_ and pH

The a*, L*, and b* values best reflect the color quality of dried meat products. In the experiment, a TCP2 type chromometer (whiteboard values of *X* = 92.18, *Y* = 95.18, and *Z* = 102.29) measured the redness. Initially, the samples were chopped and placed in the measuring container. The technical parameters used were a D65 light source, two viewing angles, and a 30‐mm condenser. Each sample was replicated three times, and the average value was used in our analyses (Xia et al., 2013). Furthermore, this study used the influence parameter *e*‐value as the main parameter to judge the color of jerky. The calculation formula for this parameter is as follows:

$$e=\frac{a^{*}}{l^{*}}+\frac{a^{*}}{b^{*}}$$

Cut an appropriate amount of samples at a consistent room temperature, place them at the bottom of the sample box, and use a fully automated water activity meter (LabSwift‐a_w_, Novasina, Switzerland) to measure the *a*_w_ value. Ten grams of camel jerky was mixed with 90 mL of double‐distilled water, and the pH value was measured with a digital pH meter (PB‐10, Sartorius, Germany).

#### 2.2.5 Sensory evaluation

Sensory perception is the first intuitive feeling consumers have regarding products and diets. Samples were assessed by a trained panel of 10 researchers from the Inner Mongolia Agricultural University using a descriptive analytic method. Selected members underwent additional training in meat and the sensory characteristics of meat products over 5 years and have since participated in several panels for jerky sensory analysis. The sensory traits and their definitions are explained in Table 3. Each evaluator was served a total of 50 samples (fermented meat jerky with different formulas and fermentation conditions). Questions were presented to assessors in the normal perception order: appearance, color, aroma, and mouthfeel. The sample order was randomized. A glass of water was provided for each assessor. All sessions were conducted in a room without peculiar smells, a temperature of 20°C, and white fluorescent lighting.

**TABLE 3:** Sensory attributes, definitions, and each attribute scored.

Sensory trait	Definition	Value
Appearance	Jerky thickness, uniform edge, and complete size	(15–20)
	The thickness of the dried meat is basically the same with some variation in the size of the broken edges	(10–14)
	The dried meat thickness is inconsistent, the edge is incomplete, and the size varies	(<10)
Color	Dried meat with an even luster of brownish red	(15–20)
	Dried meat color is basically uniform, and the glossiness is a poor brownish yellow	(10–14)
	The color of dried meat is not uniform, and the luster is black	(<10)
Aroma	The meat has a strong aroma and no burnt taste	(20–30)
	Aroma is light without any burnt taste	(10–19)
	No meat smell, but a burnt taste	(<10)
Mouthfeel	The dried meat is soft and hard, easy to chew, and has no peculiar smell	(20–30)
	The dried meat is hard and chewy with a slightly burnt taste	(10–19)
	The dried meat is very hard to chew and has an unpleasant smell	(<10)

#### 2.2.6 Determination of protein, fat, moisture, ash, phosphorus, calcium, iron, magnesium, and vitamin content

According to the Kjeldahl method outlined in the People's Republic of China National Standard GB5009.5‐2016 (2016a), 2 g of the sample was digested in a digestion furnace with copper sulfate, potassium sulfate, and sulfuric acid, and the protein content was analyzed using an automatic Kjeldahl nitrogen analyzer (Kjeltec 8100, Foss, Denmark).

According to the Soxhlet extraction method outlined in the People's Republic of China National Standard GB5009.6‐2016 (2016b), 2 g of the sample was weighed, extracted with anhydrous ether, and the fat content determined using a Soxhlet extractor.

According to the method outlined in the People's Republic of China National Standard GB5009.3‐2016 (2016c), a suitable sample quantity was placed in a drying oven at a temperature of 101–105°C. After reaching a constant weight, the moisture content was calculated.

According to the method outlined in the People's Republic of China National Standard GB5009.4‐2016 (2016d), a quantitative sample was carbonized until smokeless, then incinerated in a medium–high temperature muffle furnace at 500°C for 2–4 h to obtain dry ash, and the ash content was calculated.

Calcium, iron, and magnesium contents were determined using flame atomic absorption spectrometry (FAAS) following the People's Republic of China National Standards GB5009.92‐2016 (2016e), GB5009.90‐2016 (2016f), and GB5009.241‐2017 (2017). Molybdenum blue spectrophotometry was utilized to determine phosphorus content following the People's Republic of China National Standard GB5009.87‐2016 (2016g).

Vitamin A was quantified following the People's Republic of China National Standard GB5009.82‐2016 (2016h) utilizing reversed‐phase high‐performance liquid chromatography (HPLC). Vitamin B1 and B2 contents were assessed via fluorescence spectrophotometry according to People's Republic of China National Standards GB5009.84‐2016 (2016i) and GB5009.85‐2016 (2016j).

#### 2.2.7 Determination of amino acids

The free amino acid content was determined by automatic amino acid analyzer (L‐8900, Hitachi Limited, Japan) using the method of the People's Republic of China National Standard GB 5009.124‐2016 (2016k). Accurately weighed 30 mg of the sample dried to constant weight (accurate to 0.0001 g), added 15 mL of 6 mol/L hydrochloric acid (HCL), and hydrolyzed with the extraction temperature 90°C for 24 h. Then it was evaporated to dryness under reduced pressure with 1 mL of filtrate and fixed volume with 0.02 mol/L hydrochloric acid. It was filtered with a 0.22‐μm aperture filter and analyzed by an L‐8900 automatic amino acid analyzer.

#### 2.2.8 Determination of fatty acid content

According to O'Fallon et al. (2007), the fatty acid content was analyzed using a gas chromatography–mass spectrometry (GC–MS) system (6890N‐5975C, Agilent, USA). A 0.5 g of sample was chopped, methylated with an organic solvent, and filtered through a 0.22‐μm pore size membrane. A 1 μL sample of the filtrate was then injected into the GC–MS. An Agilent HP‐88 capillary GC column was used. The column temperature was ramped up from 120 to 230°C and held for 35 min. The injection port temperature was maintained at 250°C with a constant pressure of 190 kPa and a detector temperature of 300°C. A 1.0 μL sample was injected with a split ratio of 1:50.

#### 2.2.9 Determination of protein degradation

Following Sun's method (Sun et al., 2011), myofibrillar proteins were extracted. A 4 g sample was mixed with an extraction solution and homogenized to isolate the myofibrillar proteins. Protein concentration was measured using a bicinchoninic acid (BCA) protein assay kit. The myofibrillar protein solution was then denatured by heating with a buffer solution and loaded onto a polyacrylamide gel, consisting of an 8% separation gel and a 5% concentrated gel, for electrophoresis at a constant voltage of 100 V. After electrophoresis, the gel was stained and destained, and the bands were observed using a gel imaging system (Gel Doc XR+, Bio‐Rad, USA).

#### 2.2.10 Determination of microorganisms and TBARS

The total number of colonies was determined by agar medium culture following the People's Republic of China National Standard GB4789.2‐2022 (2022). The number of lactic acid bacteria was determined by incubation in anaerobic conditions with de Man–Rogosa–Sharpe (MRS) medium according to the method of the People's Republic of China National Standard GB4789.35‐2016 (2016l). *Escherichia coli* was determined by the plate counting method according to the People's Republic of China National Standard GB4789.3‐2016 (2016m).

The thiobarbituric acid (TBARS, mg/100 g) was quantified according to the method of Sun et al. (2022) with some modifications. Four grams of the sample was taken, 20 mL of 7.5% trichloroacetic acid (TCA) (containing 0.1 ethylenediaminetetraacetic acid (EDTA)) was added and shaken for 30 min. The mixture was filtered with double‐layer filter paper three times. Five milliliters of 0.02 mol/L thiobarbituric acid (TBA) was added to the supernatant and placed in a 90°C water bath for 40 min. It was then centrifuged at 1600 rpm (revolutions per minute) for 5 min. Five milliliters of chloroform was added to the supernatant and shaken well. After static layering, the supernatant was taken and the absorbance value was measured at 532 nm/600 nm.

### 2.3 Statistical analysis

Data analysis was conducted using SPSS 26.0 (IBM, Chicago, Illinois, USA). One‐way analysis of variance (ANOVA) was used to determine the significance of the data. Duncan's multiple range test (DMRT) was applied to evaluate differences between variables at a 5% significance level. Differences were considered significant at *p* < .05. Sensory evaluation results were analyzed using a mixed model with different treatments and sensory attributes as fixed effects and the number of sessions and the number of panels as random effects. The graphs in the article were generated with Origin 2018.

## 3 RESULTS AND DISCUSSION

### 3.1 Optimization of formula conditions for camel jerky

**FIGURE 1:**
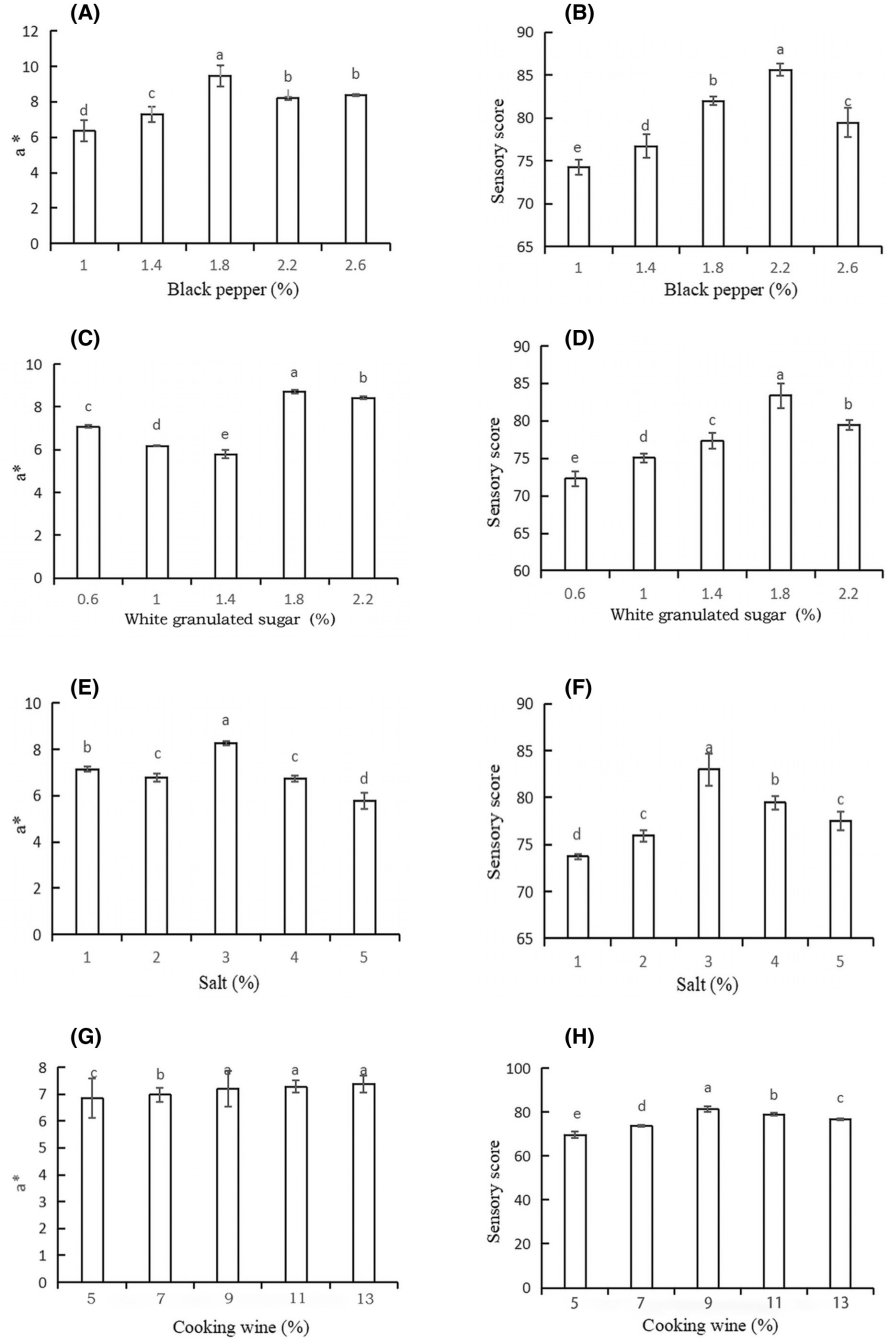
Effects of (A) black pepper, (C) white granulated sugar, (E) salt, and (G) cooking wine; supplemental amount on a* value. Effects of black pepper (B), white granulated sugar (D), salt (F), and cooking wine (H); supplemental amount on sensory perception. ^a,b,c,d,e^Mean values followed by different lowercase letters indicate significant differences.

The penetration of seasoning during curing can significantly improve the redness value a*, which is the most important chromaticity index affecting its sensory quality and can directly reflect the color of camel jerky. Therefore, the best basic formula combination was selected based on sensory quality and supplemented by the redness value a*. Figure 1A demonstrates that the addition of 1.8% black pepper significantly increased the color of camel jerky compared to other groups (*p* < .05). Black pepper has the ability to remove superoxide anion free radicals, remove hydroxyl free radicals, and resist linoleic acid lipid peroxidation. In addition, it has a good antioxidant effect and maintains the color of jerky at a certain concentration range. The sensory score of black pepper was the highest (85.61) when 2.2% was added (Figure 1B). The presence of black pepper may play a role in augmenting not only the antioxidant characteristics of the product but also its efficacy in enhancing flavor, concealing fishy odors, intensifying aroma, preserving freshness, and mitigating undesirable odors (Agbor et al., 2012). Figure 1C,D illustrates that the a* value (8.71) and the sensory score (83.37) were the highest at 1.8% white granulated sugar supplementation. White granulated sugar can also increase the flavor of meat products during the curing process. The a* value continuously increased with salt addition, and the highest sensory score was 83.01 with 3% salt addition (Figure 1E,F). Salt can promote sugar penetration into the deep layers of meat, which reduces the degree of oxidation while simultaneously slowing down the color change caused by oxygen contact with meat tissue due to the osmotic effect. Furthermore, the concentration of flavor compounds in meat products exhibits a notable association with the salt content (Lorido et al., 2015). Research conducted by Ventanas et al. has shown that salt plays a crucial role in impacting the flavor of meat products by either inhibiting or promoting lipid oxidation and protein degradation (Ventanas et al., 2010). At 9%, 11%, and 13% cooking wine, a* was significantly higher than those in the other groups, and the sensory score (81.39) was the highest at 9% cooking wine (Figure 1G,H).

The sensory score is the main index, whereas a* is the auxiliary index. The optimal orthogonal test levels were selected as follows: black pepper (1.8%, 2.2%, and 2.6%), white granulated sugar (1.4%, 1.8%, and 2.2%), salt (2%, 3%, and 4%), and cooking wine (9%, 11%, and 13%). Table S1 indicates that the sauce had certain effects on the a* and sensory properties of camel jerky. The best process combination was A_2_B_2_C_2_D_2_ according to the orthogonal experiment results, primarily based on sensory score and supplemented by the a* value. The best camel jerky formula was 2.2% black pepper, 3% salt, 1.8% white granulated sugar, and 9% cooking wine.

### 3.2 Optimization of fermentation conditions for camel jerky

In order to determine the best basic formula for camel jerky, the fermented camel jerky was prepared by adding either a single starter (*Lactobacillus plantarum* JYLP‐326) or a compound starter (*Lactobacillus plantarum* JYLP‐326 and *Pediococcus pentosaceus* JYPP‐19). Using the pH value and sensory score as evaluation indexes, the single‐factor experiment, the response surface method, and the verification experiment of the best fermentation conditions were conducted to obtain the best fermentation conditions.

**FIGURE 2:**
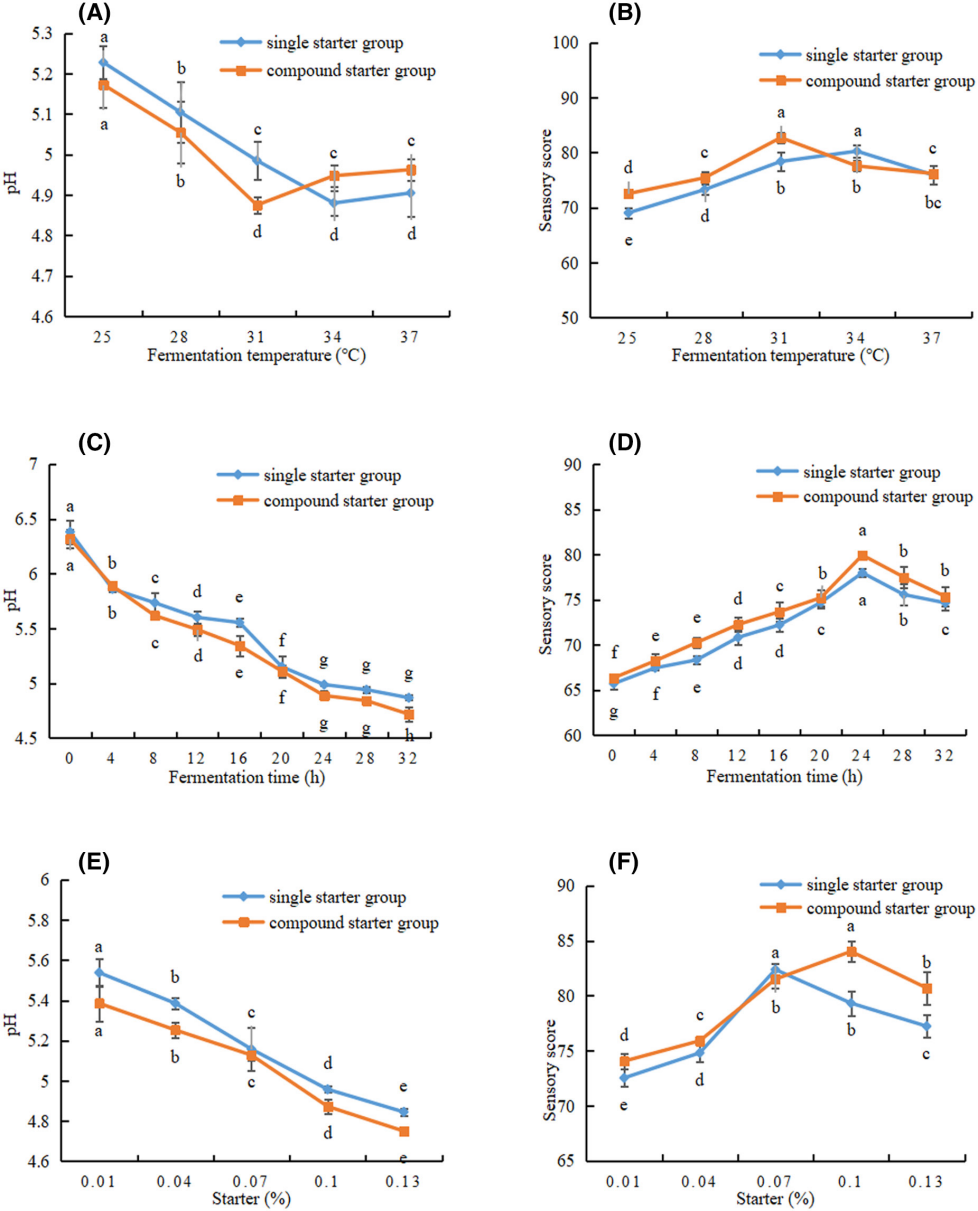
Effects of (A) fermentation temperature, (C) fermentation time, and (E) amount of starter culture on the pH. Effects of (B) fermentation temperature, (D) fermentation time, and (F) amount of starter culture on the sensory values of camel jerky fermented via two different methods. ^a,b,c,d,e^Mean values followed by different lowercase letters indicate significant differences.

As shown in Figure 2A, the pH and temperature decreased prior to increasing in both groups containing 0.10% starter culture with a fermentation time of 20 h. The pH values of the single and compound starter groups decreased to 4.96 and 4.87 at 31°C, respectively. The pH value of the compound starter group was lower than that of the single starter group at 25, 28, and 31°C, indicating that the compound starter group had strong *Lactobacillus* activity, which can reduce pH values rapidly. According to Figure 2B, the single and compound starter groups had the highest sensory scores at 34°C and 31°C, respectively. Figure 2C shows that the pH values decreased in two groups as fermentation time increased with 0.10% starter culture and fermentation temperature at 31°C. The pH gradually decreased from 24 to 32 h, indicating that it had entered the stable growth period of lactic acid bacteria. The pH decrease may be due to the accumulation of organic acids, mainly lactic acid, in the product as a result of the breakdown of carbohydrates during fermentation. The pH of the compound starter group was lower than that of the single starter group. Because the compound starter contained *Lactobacillus plantarum*, it used carbohydrates to produce a large number of acidic substances under the joint action of *Pediococcus pentosaceus*, thereby promoting the reduction of pH. Similar results were obtained by Zhao et al. (2011). In Figure 2D, the two groups had the highest sensory scores at 24 h, and the sensory score of the single starter group was lower than that of the compound starter group (77.96 vs. 79.92, respectively). According to Figure 2E, the pH value of the two groups decreased significantly with increasing starter at 31°C for 24 h (*p* < .05). The pH value of the compound starter group was lower than that of the single starter group, indicating that the acid energy of the compound starter group was superior to that of the single starter group and that the pH value could be rapidly reduced. Figure 2F reveals that the single starter group and compound starter group had the highest sensory scores at 0.07% and 0.10%, respectively.

Three optimal level groups were selected using single‐factor tests with different starter volumes (0.07%, 0.10%, and 0.13%), fermentation durations (20, 24, and 28 h), and fermentation temperatures (28, 31, and 34°C) of fermented camel jerky by single and compound starters. The regression models using these three factors were obtained through the following response surface calculations: pH single starter fermentation = 4.86 – 0.097A − 0.0776B − 0.027C + 0.075AB − 0.015 AC + 0.060 BC + 0.16A^2^ + 0.2B^2^ + 0.068C^2^. pH compound starter fermentation = 4.69 – 0.12A − 0.075B − 0.042C + 0.085AB + 5.00E − 003 AC − 0.01 BC + 0.22A^2^ + 0.17B^2^ + 0.13C^2^.

**FIGURE 3:**
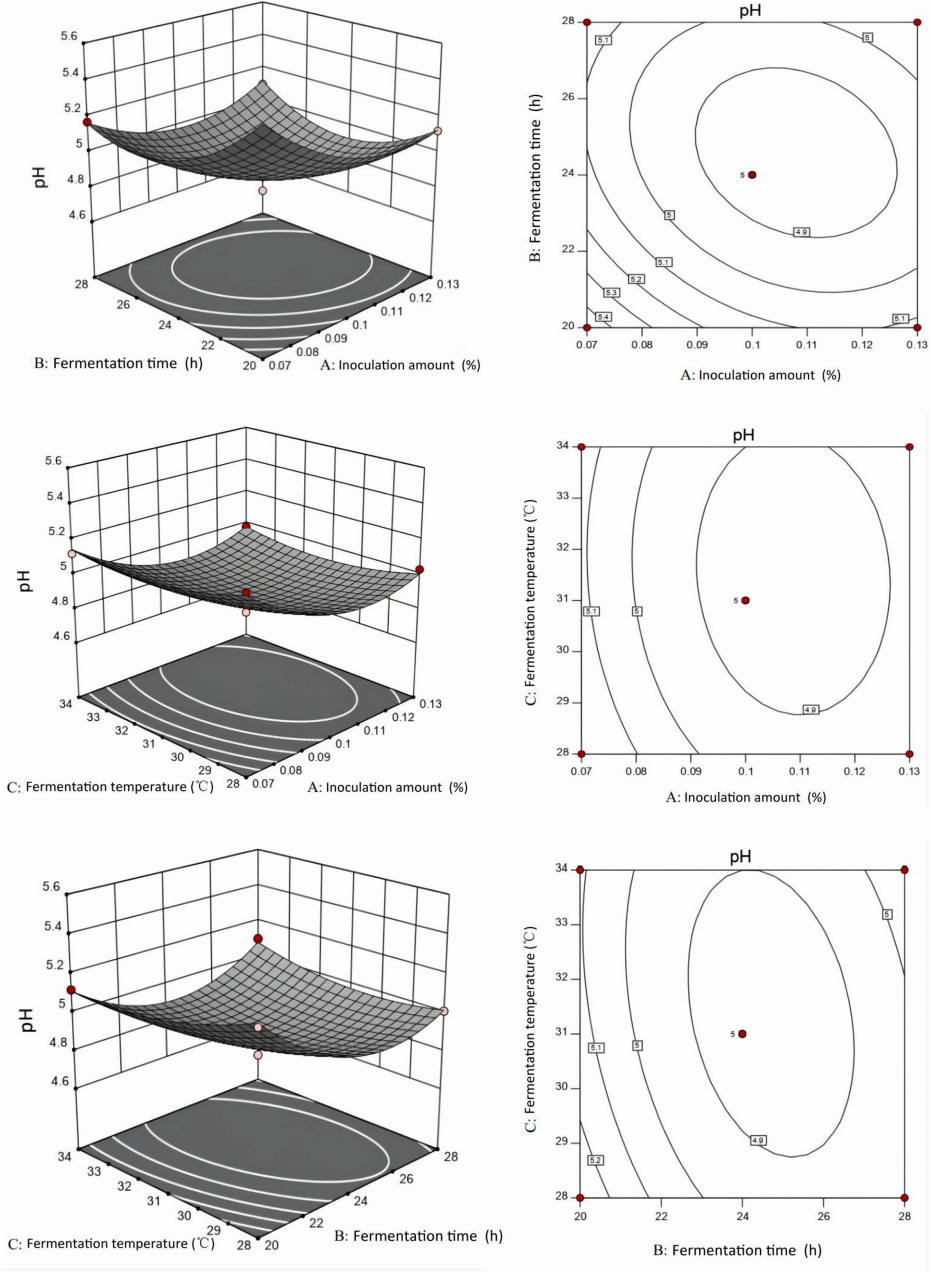
Response surface and contour map of the camel jerky fermentation using a single bacterium.

**FIGURE 4:**
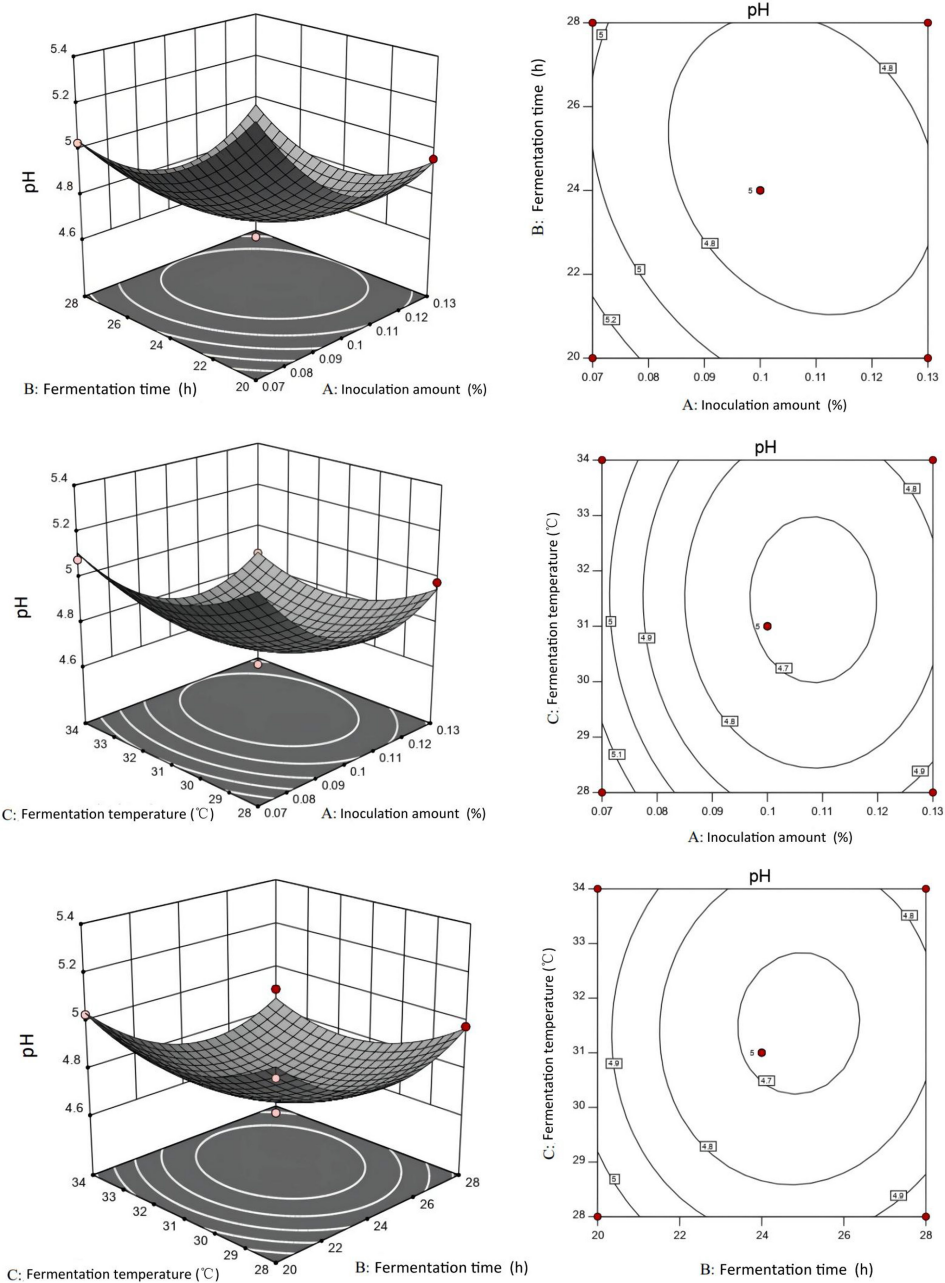
Response surface and contour map of the fermented camel jerky using a compound starter culture.

As depicted in Figures 3 and 4, the pH response value decreased significantly at 31°C as fermentation time and the amount of starter culture increased in the two groups. According to the surface slope, the response surface curve of the starter group was steeper than that of the fermentation temperature. Lactic acid bacteria utilize sugars that are metabolized into lactic acid through the glycolytic pathway, and therefore the amount of starter tends to reduce the pH response value (Zhao et al., 2011). The pH response value decreased significantly as the fermentation temperature and the amount of starter increased over 24 h, with the amount of starter having a more pronounced effect on pH than the fermentation temperature in the two groups. The pH response value decreased significantly as fermentation time and fermentation temperature increased with 0.05% single starter and compound starter, respectively; fermentation time had a greater effect on pH than fermentation temperature.

The specific experimental conditions are shown in Table 4 based on the optimization results of the two groups of fermented camel jerky. The verification test revealed that the pH value and sensory score of the fermented camel jerky produced by a compound starter culture were better than those produced by single bacteria. The optimal fermentation conditions were 0.07% starter culture for 23 h at 30°C.

**TABLE 4:** Effects of starter amount, fermentation time, and fermentation temperature on pH and sensory perception.

Item	Amount of starter (%)	Fermentation time (h)	Fermentation temperature (°C)	pH value (*M* ± SD)	Sensory score (*M* ± SD)
Single starter group	0.07	24	32	4.95 ± 0.01^a^	76.70 ± 1.85^c^
	0.08	21	30	4.91 ± 0.02^b^	75.78 ± 1.03^d^
	0.08	25	33	4.90 ± 0.02^b^	74.81 ± 1.22^d^
Compound starter group	0.07	24	31	4.86 ± 0.03^d^	83.71 ± 1.04^b^
	0.08	25	33	4.81 ± 0.02^c^	81.27 ± 0.96^b^
	0.07	23	30	4.88 ± 0.03^d^	84.48 ± 1.04^a^

*Note*: Mean values followed by different lowercase letters in the same column indicate significant differences. *M* ± SD: All results are followed by the mean ± standard deviation.

### 3.3 Comparative analysis of final product quality characteristics

Table 5 shows that the protein content of fermented camel jerky in the treatment group was significantly higher than that of the control group (*p* < .05) and that the protein content of the compound starter group was significantly higher than that of the single starter group (*p* < .05). The fat content of the compound starter group was significantly lower than that of the single starter group (*p* < .05). This could be attributed to the ability of the compound starter to increase lipase production and further degrade the fat component in camel jerky. Lactic acid bacteria can produce a large number of organic acids during fermentation, reduce the pH of jerky, and produce enzymes. Lipase can facilitate the breakdown of fat and the degradation of saturated fat, as well as impart a unique flavor and enhance the quality of fermented camel jerky (Lorenzo et al., 2014). The water content of the compound starter group was significantly lower than that of the single starter group (*p* < .05). This phenomenon aligns with the research by Visessanguan et al., which suggests that starter culture play a role in converting carbohydrates into organic acids. This process leads to the denaturation of muscle proteins and contraction of muscle bundles, causing a loss of water from the intra‐ and intermyofibrillar protein network. As a result, the water‐holding capacity of the meat product is reduced, leading to a decrease in moisture content (Visessanguan et al., 2006). The use of a compound starter culture can effectively lower water content and prevent the proliferation of harmful microorganisms (Ruiz‐Capillas & Jiménez‐Colmenero, 2004). After fermentation, the content of vitamins B_1_ and B_2_ in camel jerky increased significantly (*p* < .05), with the content of vitamin B_1_ in the compound starter group being significantly higher than that in the single starter group and the content of vitamin A in the control group being significantly higher than those in other groups (*p* < .05). The contents of calcium, magnesium, iron, and phosphorus in fermented camel jerky increased significantly (*p* < .05), indicating that *Lactobacillus* could improve the content and activity of mineral elements in the growth and metabolism processes.

**TABLE 5:** Changes in conventional composition and micronutrients during the processing of fermented jerky.

Item	Control group	Pickled group	Single starter group	Compound starter group
Protein (%)	20.75 ± 0.28^d^	21.45 ± 0.48^c^	27.71 ± 0.06^b^	29.19 ± 0.21^a^
Lipid (%)	3.14 ± 1.15^a^	2.99 ± 0.82^b^	2.83 ± 0.74^c^	2.52 ± 0.66^d^
Moisture (%)	67.46 ± 0.97^a^	35.68 ± 1.16^d^	38.16 ± 0.46^b^	36.85 ± 0.46^c^
Ash content (%)	1.24 ± 0.05^c^	3.01 ± 0.13^b^	4.13 ± 0.03^a^	4.23 ± 0.09^a^
Vitamin B_1_ (mg/100 g)	0.12 ± 0.00^c^	0.11 ± 0.01^d^	0.18 ± 0.00^b^	0.19 ± 0.01^a^
Vitamin B_2_ (mg/100 g)	0.19 ± 0.00^c^	0.25 ± 0.00^b^	0.45 ± 0.03^a^	0.47 ± 0.02^a^
Vitamin A (mg/100 g)	0.12 ± 0.00^a^	0.02 ± 0.01^b^	0.02 ± 0.00^b^	0.03 ± 0.01^b^
Ca (mg/100 g)	20.20 ± 1.77^d^	25.53 ± 2.86^c^	30.23 ± 7.05^b^	33.43 ± 3.75^a^
Mg (mg/100 g)	21.92 ± 1.06^d^	29.27 ± 0.85^c^	34.49 ± 0.61^b^	42.47 ± 0.59^a^
Fe (mg/100 g)	26.72 ± 1.08^d^	29.22 ± 0.95^c^	35.52 ± 0.84^b^	36.70 ± 1.37^a^
P (mg/100 g)	0.16 ± 0.00^b^	0.10 ± 0.02^c^	0.31 ± 0.00^a^	0.31 ± 0.01^a^

*Note*: Mean values followed by different lowercase letters in the same line indicate significant differences.

A total of 17 amino acids were detected in our amino acid composition analysis (Table 6). The total amino acid, umami substance (Asp), sweet substance (Thr), and glutamate (Glu) contents in treatment groups were higher than those in other groups (*p* > .05). This may be due to the inoculated starter culture promoting protein hydrolysis of the jerky. The proteolytic activity of lactic acid bacteria allows macromolecular proteins to be decomposed into small molecular peptides and amino acids, increasing the total free concentration of the camel jerky, which are easily digested and absorbed by the human body. During the fermentation process, the amino acids produced by *Pediococcus pentosaceus* carried a large number of delectable ingredients, which imparted the camel jerky special flavors (Aro et al., 2010). In addition, studies have shown that glutamate content is significantly increased owing to the strong proteolytic activity of *Lactobacillus plantarum* (Yang et al., 2016; Zhao, Schieber, et al., 2016).

**TABLE 6:** Changes in amino acid contents during the processing of fermented jerky.

Amino acid (%)	Control group	Pickled group	Single starter group	Compound starter group
Asp	1.73 ± 0.01^b^	1.05 ± 0.05^c^	2.75 ± 0.01^a^	2.77 ± 0.02^a^
Thr	0.84 ± 0.01^c^	1.48 ± 0.03^a^	1.33 ± 0.00^b^	1.35 ± 0.00^b^
Ser	0.72 ± 0.01^c^	1.28 ± 0.07^a^	1.10 ± 0.01^b^	1.12 ± 0.01^b^
Glu	2.91 ± 0.01^b^	2.01 ± 0.04^c^	4.48 ± 0.02^a^	4.61 ± 0.02^a^
Gly	1.11 ± 0.00^d^	1.45 ± 0.02^a^	1.24 ± 0.00^b^	1.22 ± 0.01^c^
Ala	1.15 ± 0.00^b^	1.59 ± 0.26^a^	1.65 ± 0.01^a^	1.68 ± 0.01^a^
Cys	0.24 ± 0.01^b^	0.37 ± 0.02^a^	0.38 ± 0.01^a^	0.38 ± 0.02^a^
Val	0.92 ± 0.00^c^	1.40 ± 0.06^a^	1.47 ± 0.08^b^	1.51 ± 0.05^b^
Met	0.45 ± 0.01^c^	0.78 ± 0.03^a^	0.73 ± 0.01^b^	0.76 ± 0.04^ab^
Ile	0.84 ± 0.01^b^	1.36 ± 0.15^a^	1.36 ± 0.01^a^	1.37 ± 0.01^a^
Leu	1.19 ± 0.60^b^	2.43 ± 0.21^a^	2.50 ± 0.02^a^	2.51 ± 0.01^a^
Tyr	0.66 ± 0.01^b^	1.07 ± 0.04^a^	1.06 ± 0.01^a^	1.08 ± 0.02^a^
Phe	0.76 ± 0.02^b^	1.21 ± 0.08^a^	1.24 ± 0.02^a^	1.25 ± 0.01^a^
Lys	1.61 ± 0.02^b^	2.46 ± 0.24^a^	2.38 ± 0.07^a^	2.36 ± 0.08^a^
His	0.63 ± 0.00^c^	1.19 ± 0.06^a^	1.09 ± 0.01^b^	1.08 ± 0.02^b^
Arg	1.26 ± 0.01^b^	1.10 ± 0.05^c^	1.83 ± 0.01^a^	1.82 ± 0.01^a^
Pro	0.87 ± 0.00^b^	1.13 ± 0.13^a^	1.08 ± 0.00^a^	1.08 ± 0.02^a^
AA	17.88 ± 0.64^c^	23.02 ± 1.10^b^	27.68 ± 0.17^a^	27.84 ± 0.24^a^

*Note*: Mean values followed by different lowercase letters in the same line indicate significant differences.

Unsaturated fatty acids are divided into monounsaturated fatty acids and polyunsaturated fatty acids. The oleic acid content in monounsaturated fatty acids is the highest, which has an antioxidant effect on lipoproteins. Polyunsaturated fatty acids are fatty acids that the body cannot produce on its own. They improve immunity, promote human development, regulate the expression of related genes, and reduce cardiovascular diseases. Table 7 demonstrates that the saturated fatty acid content in the control group was higher than those in the other groups, with myristic acid and palmitic acid in the single and compound starter groups being significantly lower than those in the control group (*p* < .05). Saturated fatty acids increase the risk of high cholesterol, whereas excessive palmitic acid increases the risk of cardiovascular disease. Although stearic acid is a saturated fatty acid, it can reduce low‐density lipoprotein levels (Wood et al., 2004). Unsaturated fatty acids were more prevalent in the compound starter group than in other groups. Compared with the control group, the monounsaturated fatty acid levels in the treatment groups increased. The oleic acid content of the monounsaturated fatty acids in the compound starter group was significantly higher than that in the other groups (*p* < .05). The polyunsaturated fatty acid content in the compound starter group was the highest, and the linoleic acid content was significantly higher than that in other groups (*p* < .05), indicating that the addition of a starter can effectively promote the production of fatty acids. Research shows that an increase in free fatty acids, especially unsaturated fatty acids, directly affects the sensory and nutritional qualities of fermented sausage (Gómez & Lorenzo, 2013).

**TABLE 7:** Changes in fatty acid composition and contents during the processing of fermented jerky.

Aliphatic acid (%)	Control group	Pickled group	Single starter group	Compound starter group
Lauric acid _c12:0_	0.44 ± 0.01^a^	0.35 ± 0.00^c^	0.36 ± 0.02^bc^	0.37 ± 0.00^b^
Myristic acid _c14:0_	7.54 ± 0.07^a^	5.25 ± 0.02^d^	5.68 ± 0.05^b^	5.47 ± 0.02^c^
Palmitic acid _c16:0_	31.84 ± 0.65^a^	24.33 ± 0.00^b^	24.34 ± 0.00^b^	23.83 ± 0.05^b^
Stearic acid _c18:0_	8.55 ± 0.15^c^	11.51 ± 0.02^a^	11.22 ± 0.02^b^	11.39 ± 0.02^a^
Heptadecanoic acid _c17:0_	1.32 ± 0.07^a^	1.11 ± 0.01^b^	1.11 ± 0.01^b^	1.13 ± 0.01^b^
Saturated fatty acid	49.68 ± 0.57^a^	42.55 ± 0.02^b^	42.71 ± 0.07^b^	42.19 ± 0.08^b^
Myristic acid _c14:1_	0.62 ± 0.01^a^	0.38 ± 0.00^c^	0.39 ± 0.01^c^	0.45 ± 0.06^b^
Palmitoleic acid _c16:1_	6.67 ± 0.29^a^	4.41 ± 0.03^c^	4.96 ± 0.06^b^	5.09 ± 0.02^b^
Elaidic acid _C18:1n9t_	1.51 ± 0.02^b^	1.77 ± 0.03^b^	1.9 ± 0.22^ab^	2.43 ± 0.63^a^
Oleic acid _C18:1n9c_	25.60 ± 0.45^c^	27.81 ± 0.20^b^	27.84 ± 0.54^b^	30.35 ± 0.01^a^
Eicosenoic acid _c20:1_	1.33 ± 0.03^c^	2.72 ± 0.07^a^	2.27 ± 0.01^b^	2.31 ± 0.01^b^
Monounsaturated fatty acid	35.73 ± 0.70^c^	37.08 ± 0.30^b^	37.38 ± 0.70^b^	40.63 ± −.69^a^
Linoleic acid _C18:2n6c_	5.11 ± 0.12^d^	10.34 ± 0.17^c^	11.06 ± 0.10^b^	12.96 ± 0.03^a^
Alpha‐linolenic acid _C18:3n3_	1.29 ± 0.01^b^	1.52 ± 0.25^ab^	1.56 ± 0.01^a^	1.72 ± 0.01^a^
Polyunsaturated fatty acid	6.40 ± 0.12^d^	11.86 ± 0.10^c^	12.62 ± 0.09^b^	14.69 ± 0.03^a^
Unsaturated fatty acid	42.13 ± 0.63^c^	48.94 ± 0.40^b^	50.17 ± 0.61^b^	55.31 ± 0.72^a^

*Note*: Mean values followed by different lowercase letters in the same line indicate significant differences.

**FIGURE 5:**
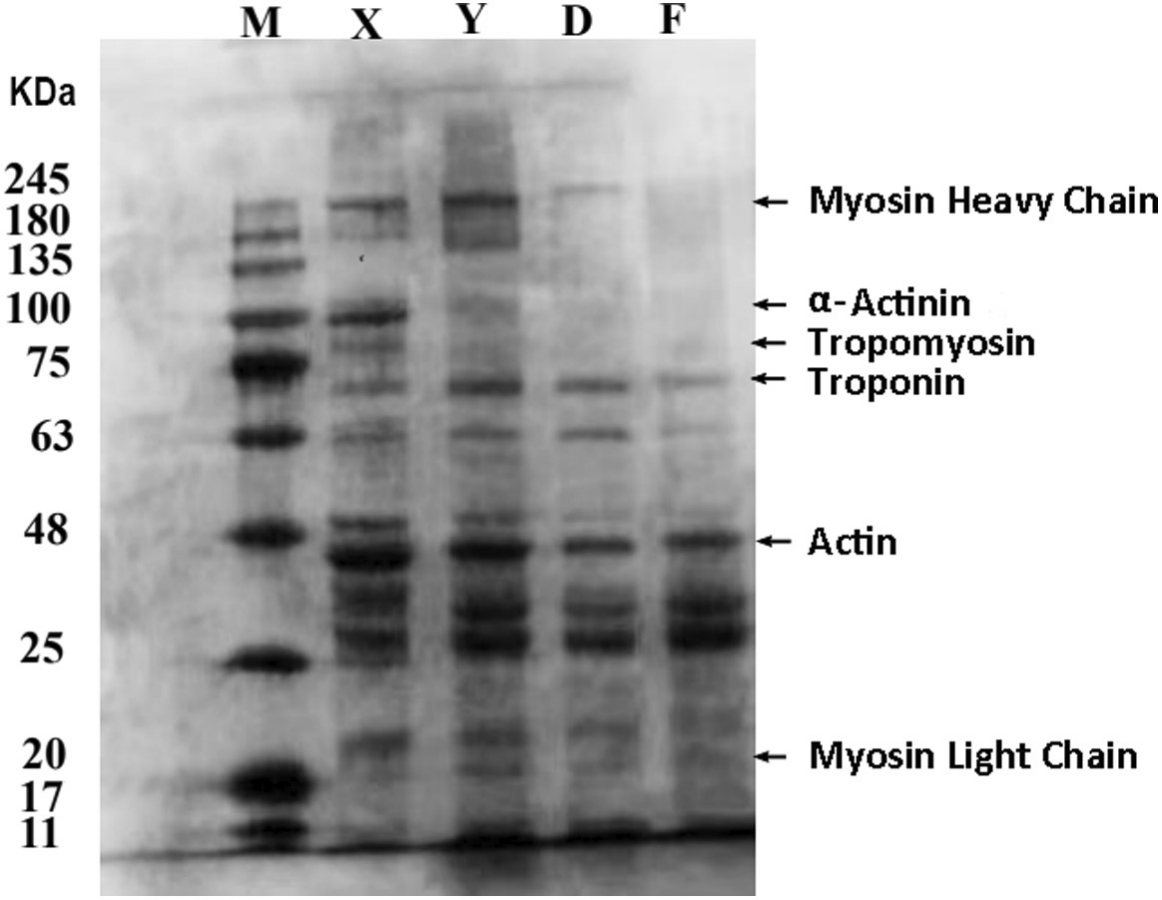
The sodium dodecyl sulfate–polyacrylamide gel electrophoresis (SDS–PAGE) analysis of camel meat and camel jerky. X: Control group; Y: Pickled group; D: Single bacteria fermentation group; F: Compound starter culture fermentation group. The first line is the Marker (M).

The characteristic bands in myofibrillar proteins are the following: myosin light chain (20 kDa), actin (45 kDa), troponin (75 kDa), tropomyosin (80 kDa), α‐auxiliary agonist protein (94 kDa), and myosin heavy chain (MHC, 220 kDa). Myofibril degradation is shown in Figure 5. The MHC, α‐coagonin (94 kDa), tropomyosin (80 kDa), and troponin (75 kDa) were significantly degraded in the compound starter group, indicating that the addition of *Lactobacillus plantarum* and *Pediococcus pentosaceus* could promote protein hydrolysis. This may be due to the decrease in pH as a result of the addition of the starter culture, whereas extreme pH induces a large amount of structural stretching of protein molecules, polymerization between protein molecules, and consequently a decrease in the solubility of the proteins. This observation aligns with the results reported by Yongsawatdigul and Hemung (2010). Researchers have found that microbial protease promotes protein decomposition and free amino acid release (Aro et al., 2010). Numerous small molecular proteins of less than 20 kDa were decomposed, and new protein molecules of 25 kDa were formed. The severe denaturation of the proteins causes solubility changes, which make it difficult to extract some molecular proteins. The macromolecular proteins in myofibril are further degraded by lactic acid bacteria, and some of the small molecular proteins are repolymerized or denatured to form new molecular proteins.

### 3.4 Comparative analysis of quality changes during storage

**FIGURE 6:**
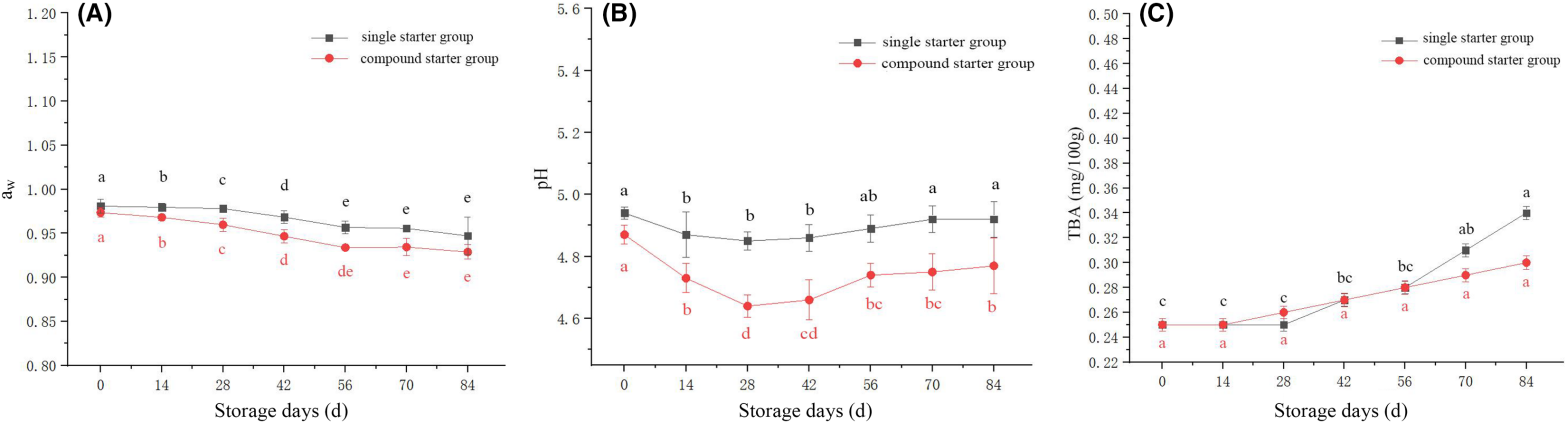
Changes in (A) *a*_w_, (B) pH, and (C) TBA of the fermented camel jerky with storage. ^a,b,c,d,e^Mean values followed by different lowercase letters indicate significant differences.

Quality alterations during storage of the camel jerky fermented with single and compound starter culture were analyzed. The measured indexes included the following: *a*_w_ value, color difference, pH, TBA, the total number of bacterial colonies, *Lactobacillus*, and *Escherichia coli*. The *a*_w_ values of the jerky samples steadily decreased with storage time (*p* < .05), and the *a*_w_ values of the compound starter group were lower than those of the single starter group. This may be due to the decrease in pH caused by the production of lactic acid during fermentation by *Lactobacillus* which leads to protein denaturation within the jerky. This alteration affects the spatial structure of the network, accelerating water loss and ultimately resulting in a rapid decrease in water content and *a*_w_ of the meat product (Fadda et al., 1999) (Figure 6A). The *a*_w_ values continuously decreased prior to 42 days (*p* < .05), but there was no evident change between 56 and 84 days of storage (*p* > .05). *a*_w_ values can ensure the stability of microorganisms during storage. Reducing *a*_w_ values can improve textural characteristics and have a significant impact on the flavor and color of the product (Jr. Fontana, 2000). The redness value a*, brightness value L*, and *e*‐value were higher in the compound starter group than in the single starter group. The redness value a* is the main color index that affects the sensory quality of fermented camel jerky, and the brightness value L* and yellowness value b* have certain impacts on jerky color. The pH value of camel jerky decreased to less than 5.0, and the pH value of the compound starter group was lower than that of the single starter group (Figure 6B). The pH values of the two samples initially decreased and then increased, which was owing to the reverse inhibition of the formation of lactic acid after the acidity decreased to a certain extent. In addition, the lactic acid bacteria can also cause a gradual increase in pH by decomposing small molecular alkaline ammonia substances, nitrogen, and protein‐derived free amino acids (Sun et al., 2022). This process has a certain inhibitory effect on the metabolic activity of the starter, ultimately impacting the overall metabolic activity level. During this period, the enzyme activity of ammonia‐producing alkaline substances becomes more active (Özyurt et al., 2009). During storage, the TBA of camel jerky fermented by a compound starter culture was lower than that of camel jerky fermented by a single bacterium (Figure 6C). However, the TBA growth rate was slow and did not exceed the safe range. The total number of bacterial colonies and lactic acid bacteria in the compound starter group was greater than that in the single starter group.

In contrast, there was no significant difference in the change of a* in the treatment group during storage (*p* > .05) (Table S2). However, the a* of the compound starter group was slightly higher than that of the single starter group. This difference may be attributed to the nitrate reductase enzyme produced by *Pediococcus pentosaceus* in the compound starter, which catalyzes the conversion of nitrite to nitrogen oxides. Subsequently, these oxides react with myoglobin present in the meat, facilitating the formation of nitro‐myoglobin and ultimately resulting in a stable red color (Baka et al., 2011; Qi et al., 2021). In addition, the L* and *e* values of the compound starter group were higher than those of the single starter group. Throughout the entire storage period, the b* of the single and compound starter groups changed marginally, and the differences were not statistically significant (*p* > .05).

During storage, no *Escherichia coli* was detected in the two groups. Kaban discovered that the compound starter group clearly inhibits *Escherichia coli* (Kaban & Kaya, 2006). In addition, *Pediococcus pentosaceus* is capable of inhibiting pathogenic and harmful bacteria, improving product hygiene indicators, and extending shelf life. Table 8 shows that the total number of colonies in the compound starter group exceeded that in the single starter group. The total number of colonies was 6.47 and 5.38 cfu/g at 0 days in the compound and single starter groups, respectively. The total number of bacteria in the treatment group increased significantly with an increase in storage days (*p* < .05). With an increase in storage days, the number of lactic acid bacteria in the compound starter group was higher than that in the single starter group. The number of lactic acid bacteria in the single starter group increased significantly between 0 and 56 days of storage (*p* < .05) and then decreased significantly (*p* < .05). The number of lactic acid bacteria in the compound starter group increased significantly from 0 to 70 days of storage (*p* < .05) and then decreased without any significance (*p* > .05). When the acidity level reaches a specific threshold, it is possible that it may inhibit the growth of lactic acid bacteria and consequently abbreviate the fermentation process (Bao et al., 2018; Liu et al., 2014).

**TABLE 8:** Effect of single starter cultures and compound starter cultures on the total number of bacteria and *Lactobacillus* (cfu/g) of fermented jerky during storage.

Storage days (days)	Total number of bacteria		*Lactobacillus*	
	Single starter group	Compound starter group	Single starter group	Compound starter group
0	5.38 ± 0.01^d^	6.47 ± 0.06^e^	5.29 ± 0.07^e^	6.36 ± 0.12^e^
14	5.03 ± 0.06^e^	7.01 ± 0.03^d^	5.49 ± 0.03^d^	7.06 ± 0.02^d^
28	5.54 ± 0.02^c^	7.03 ± 0.04^d^	5.66 ± 0.02^c^	7.10 ± 0.01^c^
42	5.53 ± 0.01^c^	7.07 ± 0.01^c^	5.72 ± 0.01^c^	7.13 ± 0.05^c^
56	5.69 ± 0.02^b^	7.12 ± 0.05^b^	5.87 ± 0.02^a^	7.20 ± 0.02^b^
70	5.81 ± 0.03^a^	7.19 ± 0.02^a^	5.85 ± 0.02^b^	7.26 ± 0.02^a^
84	5.83 ± 0.01^a^	7.18 ± 0.02^a^	5.81 ± 0.04^b^	7.25 ± 0.03^a^

*Note*: Mean values followed by different lowercase letters in the same column indicate significant differences.

## 4 CONCLUSIONS

In this study, response surface experiments, verification tests, and multiple comparative analyses were performed to determine the nutritional benefits of new camel jerky developed using modern fermentation technology. In summary, the optimum basic parameters for camel jerky preparation were black pepper (2.2%), salt (3%), sugar (1.8%), and cooking wine (9%). The optimal fermentation conditions were as follows: the addition of the starter (0.07%) and the fermentation of meat for 23 h at 30°C. The compound starter culture fermentation of camel jerky showed remarkable characteristics, including a high nutritional composition, a good taste and texture, and a high level of stability during storage. The compound starter (*Lactobacillus plantarum* JYLP‐326 and *Pediococcus pentosaceus* JYPP‐19) of camel meat can be used for the development of a new meat‐based health food product in the industry.

## AUTHOR CONTRIBUTIONS

**Jindi Wu:** Conceptualization (equal); data curation (equal); formal analysis (equal); funding acquisition (equal); investigation (equal); methodology (equal); software (equal); validation (equal); writing – original draft (equal); writing – review and editing (equal). **Xige He:** Conceptualization (equal); data curation (equal); formal analysis (equal); investigation (equal); methodology (equal); software (equal); validation (equal); visualization (equal); writing – original draft (equal); writing – review and editing (equal). **Xueyan Yun:** Formal analysis (equal); investigation (equal); software (equal). **Mei Qi:** Data curation (equal); formal analysis (equal); investigation (equal); software (equal); validation (equal); visualization (equal). **Buren Menghe:** Resources (equal); supervision (supporting). **Lu Chen:** Formal analysis (equal); investigation (equal); software (equal); validation (equal); visualization (equal). **Yunfei Han:** Investigation (equal); software (equal); validation (equal); visualization (equal). **Yajuan Huang:** Investigation (supporting); software (supporting); visualization (supporting). **Mingxu Wang:** Investigation (supporting); software (supporting); validation (supporting). **Rina Sha:** Data curation (supporting); investigation (supporting); writing – review and editing (supporting). **Gerelt Borjigin:** Conceptualization (equal); funding acquisition (lead); project administration (lead); resources (lead); supervision (lead); writing – review and editing (supporting).

## FUNDING INFORMATION

This work was supported by the Inner Mongolia Autonomous Region Science and Technology Plan Project (2019–2022), the China Agriculture Research System of the MOF and the MARA (Grant No. CARS‐38), and the Natural Science Foundation of Inner Mongolia (Grant No. 2022QN03026).

## CONFLICT OF INTEREST STATEMENT

None.

## ETHICS STATEMENT

This study complied with the Laboratory Animal—Guideline for Ethical Review of Animal Welfare (National Technical Committee on Laboratory Animals of Standardization Administration of China, 2018) and approved by the Specialized Committee on Scientific Research and Academic Ethics of Inner Mongolia Agricultural University (approval document number [2020]002, dated April 7, 2020).

## Supporting information


Table S1

Table S2

## Data Availability

The data presented in this study are available on request from the corresponding author.
